# CSF-1 and Notch signaling cooperate in macrophage instruction and tissue repair during peripheral limb ischemia

**DOI:** 10.3389/fimmu.2023.1240327

**Published:** 2023-08-24

**Authors:** Tamar Kapanadze, Jaba Gamrekelashvili, Stefan Sablotny, Dustin Kijas, Hermann Haller, Kai Schmidt-Ott, Florian P. Limbourg

**Affiliations:** ^1^ Vascular Medicine Research, Hannover Medical School, Hannover, Germany; ^2^ Department of Nephrology and Hypertension, Hannover Medical School, Hannover, Germany

**Keywords:** macrophages, ischemia, inflammation, CSF-1, notch signaling, CSF-1 inhibition

## Abstract

Ischemia causes an inflammatory response featuring monocyte-derived macrophages (MF) involved in angiogenesis and tissue repair. Angiogenesis and ischemic macrophage differentiation are regulated by Notch signaling *via* Notch ligand Delta-like 1 (Dll1). Colony stimulating factor 1 (CSF-1) is an essential MF lineage factor, but its role in ischemic macrophage development and the interaction with Notch signaling is so far unclear. Using a mouse model of hind limb ischemia with CSF-1 inhibitor studies and Dll1 heterozygous mice we show that CSF-1 is induced in the ischemic niche by a subpopulation of stromal cells expressing podoplanin, which was paralleled by the development of ischemic macrophages. Inhibition of CSF-1 signaling with small molecules or blocking antibodies impaired macrophage differentiation but prolonged the inflammatory response, resulting in impaired perfusion recovery and tissue regeneration. Yet, despite high levels of CSF-1, macrophage maturation and perfusion recovery were impaired in mice with *Dll1* haploinsufficiency, while inflammation was exaggerated. *In vitro*, CSF-1 was not sufficient to induce full MF differentiation from donor monocytes in the absence of recombinant DLL1, while the presence of DLL1 in a dose-dependent manner stimulated MF differentiation in combination with CSF-1. Thus, CSF-1 is an ischemic niche factor that cooperates with Notch signaling in a non-redundant fashion to instruct macrophage cell fate and maturation, which is required for ischemic perfusion recovery and tissue repair.

## Introduction

Acute or chronic limb ischemia, usually caused by peripheral artery disease (PAD) due to atherosclerosis, is an important health burden worldwide. It is associated with impaired quality of life, limb amputation and high risk for further cardiovascular complications, including myocardial infarction and stroke (1, 2). The mouse hind limb ischemia (HLI) model is a well-known animal model of PAD, recapitulating key aspects of ischemia, inflammation and arteriogenesis or neovascularization found in human patients (3).

Ischemia and the resultant hypoxia lead to cellular oxidative stress and remodeling of oxygen metabolism, resulting into cell death, release of cytokines, chemokines and “danger molecules” (4). Hypoxia and oxidative stress induce activation of hypoxia-inducible factor HIF-1α, which induces expression of VEGF, an activator of neovascularization (5). Release of chemokines in the ischemic tissue attracts inflammatory immune cells involved in tissue injury, but also angio- and arteriogenesis and subsequent tissue healing (6).

Ly6C^hi^ classical”, or “inflammatory” monocytes (Ly6C^hi^ Mo) are recruited transiently to muscle tissue after induction of HLI in mice, which is regulated by the CCR2/CCL2 axis (7–9). Controlled by the local inflammatory milieu, Ly6C^hi^ monocytes differentiate into ischemic macrophages (8) (MF), which play a crucial role in the restoration of muscle perfusion and tissue healing by promoting angio- and arteriogenesis (8, 10–12). Furthermore, in the setting of muscle injury, macrophages are involved in clearance of tissue debris (13) and induction of satellite cell proliferation during formation of regenerative muscle fibers (14).

Macrophage colony stimulating factor, also known as CSF-1, is a key myeloid lineage factor promoting development, differentiation and survival of mononuclear phagocytic cells (15, 16). CSF-1 production is increased during inflammation, including ischemia (17–21), which drives monocyte to macrophage conversion, macrophage proliferation, maturation and migration (13, 21–25). However, the role of CSF-1 for ischemic macrophage development during skeletal muscle ischemia is largely unknown.

CSF-1 is secreted by various cell types, such as blood vessel endothelial cells (EC) and mesenchymal stromal cells (26), lymphatic endothelial cells (LEC) (27, 28), fibroblasts (29) and neurons (30). However, CSF-1 expression is often localized to specific organized cellular milieus known as niches (31), and niche-specific depletion of CSF-1 leads to elimination of local resident macrophage subsets (28). The architecture and role of the niche, however, is not limited to CSF-1 production from these “nurturing” cells but rather implies a dual interaction between CSF-1 producers and macrophages (29). In line with this, the niche also contains other cell types, providing different soluble factors or contact-dependent signals which imprint macrophages to obtain tissue- and condition-specific identity (31).

Notch signaling, activated by cell-bound ligands, is involved in cell-fate decisions of hematopoietic cells including monocytes and macrophages (32–34). Notch ligands Dll1 and Dll4 are important niche-specific factors involved in resident macrophage development and maturation (35–37). In skeletal muscle, Dll1 is expressed by vascular EC and upregulated during ischemia (38), which is required for functional differentiation of recruited Ly6C^hi^ monocytes into macrophages with reparative functions. Dll1-primed macrophages are highly phagocytic and have reduced proliferation potential, demonstrating a gene expression profile and phenotype characteristic of terminally differentiated cells. Furthermore, priming of macrophages with Dll1 is critical to obtain pro-angiogenic functions and restore neovascularization and perfusion after ischemia (8).

We hypothesized that Dll1 expressing EC are part of a functional niche for monocyte-macrophage differentiation, in which Dll1 and CSF-1 cooperate. We employed a mouse model of hind limb ischemia in combination with CSF-1 inhibition or genetic Dll1 haploinsufficiency. We show that CSF-1 is specifically produced by a subpopulation of PDPN^+^ stromal cells in ischemic muscle and that CSF-1 is required but not sufficient to instruct ischemic macrophage differentiation. Instead, CSF-1 and Dll1 act in concert in a non-redundant fashion to instruct ischemic macrophage maturation.

## Results

### Induction of CSF-1 in the ischemic muscle niche correlates with development of ischemic macrophages.

**Figure 1:**
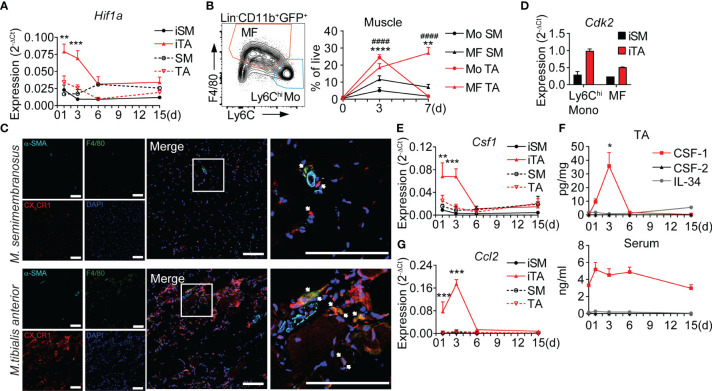
Hypoxia triggers local secretion of CSF-1 and accumulation of monocytes and macrophages in ischemic muscle. **(A)** Time-lapse analysis of *Hif1a* expression. RNA was isolated from ischemic muscles (iSM and iTA) and non-ischemic (contralateral) SM and TA muscles, n=4/5/5/6 mice, cumulative from N=2-3 independent experiments, 2-way ANOVA with Tukey’s multiple comparison test, **P<0.01, ***P<0.001. **(B)** Representative flow cytometry graph of gated cells isolated from iTA of *Cx3cr1^gfp/+^* mice, at d3 after HLI and gates for monocytes (CD11b^+^GFP^+^Ly6C^hi^F4/80^-^) and MF (CD11b^+^GFP^+^Ly6C^lo/neg^F4/80^+^) (left) and cell frequencies in the SM and TA muscles (right) before (d0) and d3/d7 after induction of ischemia. n=2/12/13 mice cumulative from N=3 independent experiments, 2-way ANOVA with Tukey’s multiple comparison test, **P<0.01, ^####/^****P<0.001. (*) indicates the difference between groups in TA muscle, (^#^) corresponds to comparison between SM and TA. **(C)** Representative laser scanning micrograph of iTA and iSM muscle sections at d3 of HLI. x200 original magnification, scale bar=100μM. Ultimate right graph shows 4x zoom of the marked area. Arrows indicate monocytes (red) and macrophages (yellow). **(D)** Expression of *Cdk2*, RNA was isolated from Ly6C^hi^ monocytes and MF sorted from the iSM and iTA muscles at d3 after HLI. Data are representative of 2 independent experiments. **(E)** Time-lapse analysis of *Csf1* expression, RNA was isolated from iSM, iTA and non-ischemic (contralateral) SM and TA muscles, n=4/5/5/6 mice, cumulative from N=2-3 independent experiments, 2-way ANOVA with Tukey’s multiple comparison test, **P<0.01, ***P<0.001. **(F)** Quantitative analysis of CSF-1, CSF-2 and IL-34 in TA muscles, n=2/6/5/3/3, cumulative from N=2-3 independent experiments; *P<0.05, One-way ANOVA with Dunnett’s multiple comparison test. d0 corresponds to not operated mouse TA. **(G)** Time-course of *Ccl2* expression, RNA was isolated from iSM, iTA and non-ischemic contralateral SM and TA muscles, N=4/5/5/6 mice, cumulative from N=2-3 independent experiments, 2-way ANOVA with Tukey’s multiple comparison test, ***P<0.001. SM, semimembranosus muscle; TA, tibialis anterior muscle.

The ischemic tissue response consists of distinct vascular, metabolic and inflammatory components organized in a spatio-temporal fashion, which can be analyzed in the HLI model (9). To further characterize the regional ischemic response in the upper vs. the lower limbs we performed comparative analysis of the proximal semimembranosus (SM) and distal tibialis anterior (TA) muscles. Following HLI, hypoxia-inducible factor *Hif1a* expression increased rapidly and significantly only in the distal TA muscle, but not in the proximal SM muscle, which never increased to levels above contralateral limb (**Figure 1A**), suggesting a hypoxic niche in the distal limb muscle, but not in the proximal limb muscle of the same leg. The rapid induction of hypoxia in TA muscle was accompanied by robust but transient recruitment of monocytes, which differentiate into a sustained population of macrophages (8) (**Table 1**), while both populations showed only moderate and transient increases in SM muscle (**Figure 1B**). By histology, few CX_3_CR1^+^ monocytes and CX_3_CR1^+^F4/80^+^ macrophages were detected in between muscle fibers or surrounding collateral arteries (α-SMA^+^) in ischemic SM (iSM). In comparison, while the pattern of localization was maintained in ischemic TA muscle (iTA), cell abundance was remarkably higher (**Figure 1C**). Interestingly, monocytes and macrophages from iTA also expressed higher levels of cyclin-dependent kinase *Cdk2* (**Figure 1D**), suggesting higher proliferation activity in the ischemic niche.

**Table 1:** Definition of cell phenotypes.

Phenotypes	Cell type
CD45^+^Lin^-^CD11b^+^F4/80^-^CX_3_CR1^+^Ly6C^hi^	Ly6C^hi^ Monocytes
CD45^+^Lin^-^CD11b^+^F4/80^-^CX_3_CR1^+^Ly6C^lo/neg^CD11c^+^CD43^+^	Ly6C^lo^ Monocytes
CD45^+^Lin^-^CD11b^+^F4/80^+^CX_3_CR1^+^Ly6C^lo/neg^	Macrophages
CD45^+^Lin^-^CD11b^+^CX_3_CR1^+^F4/80^-^Ly6C^lo/neg^CD11c^hi^Ia^+^	CX_3_CR1^+^ DC
CD45^+^Lin^+^CD11b^+^CX_3_CR1^-^Ly6C^int^SSC^int^FCS^hi^	Neutrophils
CD45^-^Ter119^-^CD31^+^PDPN^-^	EC
CD45^-^Ter119^-^CD31^+^PDPN^+^	LEC
CD45^-^Ter119^-^CD31^-^PDPN^+^	PDPN^+^
CD45^-^Ter119^-^CD31^-^PDPN^-^	DN

Lin=B220/CD19/Ly6G/CD3/NK1.1/Ter-119.

(in *Cx3cr1^gfp/+^* mice, GFP fluorescence reflects *Cx3cr1* expression).

To analyze the associated growth factor milieu, we measured key myeloid growth factors. Expression of macrophage colony-stimulating factor *Csf1* increased rapidly after induction of HLI and maintained high levels until d3, paralleling the peak in monocyte recruitment and the development of ischemic macrophages, while expression of *Csf1* in iSM did not increase over time (**Figure 1E**). Protein levels of CSF-1 in iTA showed similar dynamics, with an expression peak on d3, but serum levels of CSF-1 did not change significantly over time (**Figure 1F**), indicating local ischemic production. In contrast, levels of myeloid growth factors CSF-2 and IL-34 remained low in iTA and serum throughout the time course after HLI (**Figure 1F**). Thus, the ischemic muscle niche is characterized by specific expression of *Csf1*, suggesting an important role in regulating the ischemic inflammatory response. Interestingly, dynamics of CSF-1 on gene expression and protein levels was correlating with the dynamics of *Ccl2* expression (**Figure 1G**), and both were coinciding with the peak of Ly6C^hi^ monocytes and the development of macrophages (d3). These data indicate an early and specific ischemic niche response, promoting accumulation of Ly6C^hi^ monocytes and their conversion into macrophages (8).

### A PDPN^+^ cell population expresses CSF-1 in the ischemic niche

**Figure 2:**
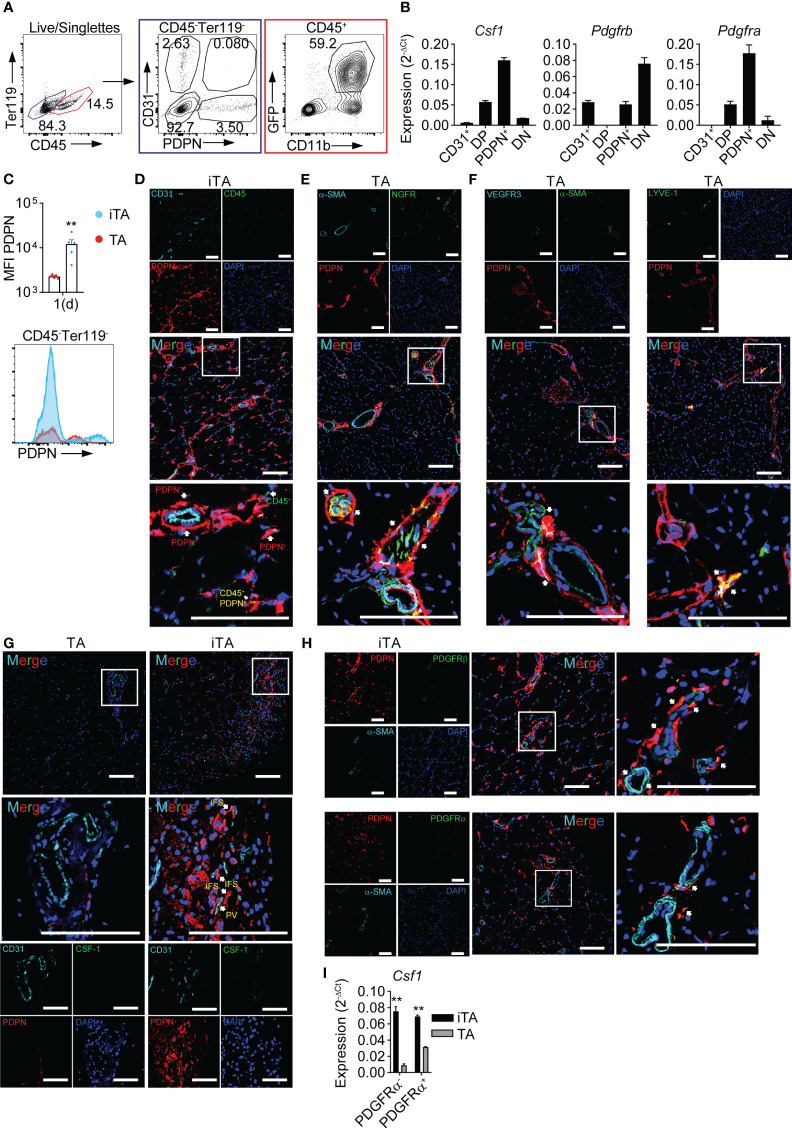
PDPN^+^ cells produce CSF-1 in the ischemic muscle. **(A)** Representative flow cytometry plot showing gating strategy used for cell sorting from ischemic muscles. **(B)** Gene expression analysis; RNA was isolated from CD45^-^ cell populations sorted from iTA muscle at d1 of HLI; data are representative of N=3 independent experiments. **(C)** Expression of PDPN on CD45^-^ cells of iTA and TA muscles at d1 after HLI induction. Data show: pool of MFI values from N=2 independent experiments (upper graph), **P<0.01, Mann-Whitney’s unpaired test and representative flow cytometry plot (lower graph). **(D–H)** Representative confocal laser scanning micrographs of frozen **(D–F, H)** and paraffin-embedded **(G)** TA and d1 iTA muscle sections. Original magnification: x200, magnification of boxed area: 4x zoom, Scale bar=100μM. Arrows: **(D)** PDPN^+^ cells (red), CD45^+^ cells (green) and CD45^+^PDPN^+^ cells (yellow); **(E)** nerve sheaths (PDPN^+^, red) and nerve fibers (PDPN^+^NGFR^+^, yellow); **(F)** lymph vessels: PDPN^+^VEGFR3^+^ (white) and PDPN^+^LYVE-1^+^ (yellow); **(G)** PDPN^+^CSF-1^+^ cells (yellow) are located in the interfiber (IFS) and perivascular (PV) space; **(H)** perivascular PDPN^+^PDGFRβ^+^ and PDPN^+^PDGFRα^+^ cells (both yellow). **(I)** Analysis of *Csf1* expression; RNA was isolated from PDPN^+^PDGFRα^-^ and PDPN^+^PDGFRα^+^ cells, sorted from TA and iTA muscles at d1 after HLI. Data are representative of N=2 independent experiments. **P<0.01 unpaired t-test.

To determine the muscle-resident cell types producing CSF-1 during ischemia we performed cell sorting and gene expression analysis, employing cell type specific markers CD45 and CD31 in combination with podoplanin (PDPN), a marker for stromal cell populations (39). We thus defined vascular EC (Ter119^-^, CD45^-^, CD31^+^), lymphatic EC [Ter119^-^, CD45^-^, CD31^+^PDPN^+^, i.e., double positive (DP)], PDPN^+^ stromal cells (Ter119^-^, CD45^-^) and double negative (DN) CD31^-^PDPN^-^ cells (**Figure 2A**). In gene expression analysis, PDPN^+^ stromal cells showed the highest expression of *Csf1*, followed by lower expression in DP cells, while vascular EC and DN cells did not show relevant levels of expression. Furthermore, PDPN^+^ stromal cells expressed high levels of *Vim*, *Col1* and *Pdgfra*, but low levels of *Pdgfrb*, consistent with fibroblasts (40) (**Figure 2B**, **Supplementary Figure 1A**). Interestingly, PDPN surface expression on CD45^-^ cells isolated from iTA muscle increased during ischemia (**Figure 2C**). To characterize PDPN^+^ cell types we performed tissue staining and fluorescence microscopy. Consistent with a stromal fibroblast population, PDPN^+^ cells surrounded blood vessels and were scattered in between muscle fibers (interfiber space, IFS) (**Figure 2D**), were found around and within nerve fibers (**Figure 2E**) or were co-expressing LYVE-1/VEGFR3 in tubular structures (**Figure 2F**), consistent with lymph vessels. In line with flow cytometry data, microscopy also demonstrated upregulation of PDPN expression in ischemic tissue (**Figure 2D** versus **Figures 2E, F**). Along with CD45^-^ cells, PDPN was also found on CD45^+^ cells (**Figure 2D**). Furthermore, spatial analysis revealed that CSF-1 expression co-localized within PDPN^+^ cells located in the proximity of blood vessels in iTA muscle, while no expression was detected in contralateral TA muscle (**Figure 2G**). Expression of PDFGRα and PDFGRβ also were found in perivascular (PV) PDPN^+^ cells (**Figure 2H**). However, after separation of PDPN^+^PDFGRα^+^ and PDPN^+^PDFGRα^-^ subpopulations (**Supplementary Figure 1B**), both demonstrated *Csf1* expression, which excluded PDFGRα as a general marker for definition of CSF-1 producing PDPN^+^ cells (**Figure 2I**).

Along with *Csf1*, ischemia also induced expression of *Ccl2*, a mediator of monocyte recruitment. Although PDPN^+^ cells showed the highest level of *Ccl2* expression, EC, LEC and DN cells also showed detectable levels (**Supplementary Figure 1C**). Of note, GFP^+^CD11b^+^ cells isolated from *Cx3cr1^gfp/+^* reporter mice (**Figure 2A**), comprising monocytes and macrophages (8) expressed inflammatory cytokines, *Tnfa*, *Il1b* and *Il6*, but also expressed *Ccl2* (**Supplementary Figure 1D**), potentially contributing to recruitment of circulating monocytes. In contrast, these cells did not express relevant levels of *Csf1* (**Supplementary Figure 1D**). Separating PDPN^+^ cells into PDGFRα cell subsets revealed only quantitative, but not principle, differences in *Ccl2* expression (**Supplementary Figure 1E**).

### CSF-1 inhibition impairs macrophage maturation and ischemic tissue recovery

**Figure 3:**
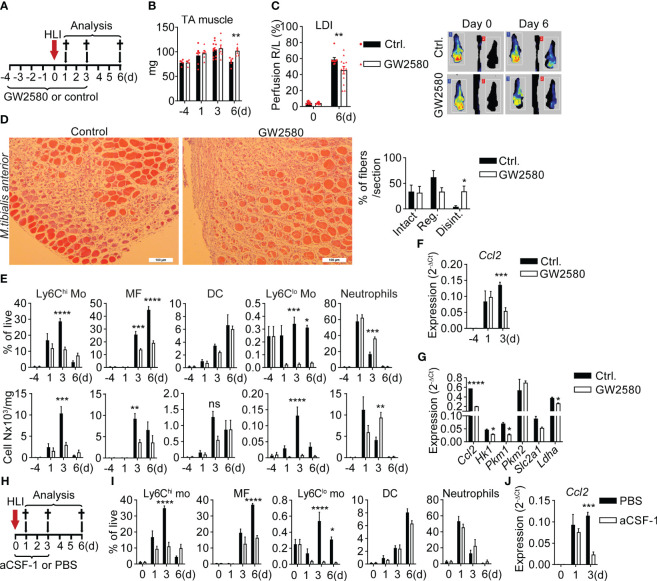
CSF-1 inhibition impairs macrophage maturation and ischemic tissue recovery **(A–H)** Administration of CSF-1 signaling inhibitor GW2580 in *Cx3cr1^gfp/+^* mice. **(A)** Experimental setup of GW2580 administration. **(B)** Mass of TA muscle in non-operated mice (d0) and after induction of HLI. n=4/6/11/5 (ctrl.) and n=4/5/7/6 (GW2580) mice. **P<0.01 Unpaired t-test. **(C)** Footpad perfusion was measured using Laser Doppler Imaging (LDI) immediately after induction of HLI (d0) and at a d6. Data show pooled results of n=9 (ctrl.) of n=12 (GW2580) mice and representative color-coded LD images for each group. **P<0.01, 2-way ANOVA with Bonferroni’s multiple comparison test. **(D)** Representative H&E images of TA muscle sections from ctrl. and GW2580-fed mice at d6 (left) and quantification of fibers (right). n=5 mice/group. *P=0.02, unpaired t-test **(E)** Time-lapse analysis of frequencies (upper panel) and absolute numbers (lower panel) of myeloid cells in the TA muscle of ctrl.- and GW2580-fed mice, n=3/6/11/5 (ctrl.) and n=3/5/6/6 (GW2580). *P<0.05, **P<0.01, ***P<0.001, ****P<0.0001. 2-way ANOVA with Tukey’s multiple comparison test. **(F)** Expression of *Ccl2*, RNA was isolated from TA muscle before (d0) and after induction of ischemia, n=3/3/9 (ctrl.) and 3/4/7 (GW2580). ***P<0.001, 2-way ANOVA with Tukey’s multiple comparison test. **(G)** Gene expression analysis, RNA was isolated from CD11b^+^GFP^+^ cells, sorted from the iTA muscles of ctrl. and GW2580-fed *Cx3cr1^gfp/+^* mice at d3 after induction of HLI. Representative of N=2 independent experiments is shown, *P<0.05, ****P<0.0001, Unpaired t-test. **(H–J)** blockade of CSF-1 by subcutaneous application of aCSF-1 antibody in the ischemic limb. Data show **(H)** experimental setup, **(I)** relative frequencies of myeloid cells, n=3/3/4/4 (ctrl.) and n=3/3/5/6 (aCSF-1) mice, *P<0.05, ****P<0.0001. 2-way ANOVA with Tukey’s multiple comparison test. **(J)** Expression of *Ccl2*, RNA was isolated from the TA muscle, n=3/3/4 (ctrl.) and n=3/3/5 (aCSF-1) mice, ***P<0.001. 2-way ANOVA with Tukey’s multiple comparison test.

To determine the functional role of CSF-1 in the ischemic niche we next inhibited CSF-1 actions. CSF-1 signals through cFMS receptor tyrosine kinase (41), which is inhibited by the specific cFMS receptor tyrosine kinase inhibitors GW2580 (42). *Per os* treatment with GW2580 (**Figure 3A**) resulted in delayed normalization of TA muscle edema, measured as muscle mass (**Figure 3B**), and delayed distal limb perfusion recovery by Laser Doppler imaging (LDI, **Figure 3C**), while *Csf1* expression in GW2580 treated mice was slightly increased (**Supplementary Figure 2A**). To quantify muscle damage and regeneration we analyzed three distinct features of muscle fibers: shape, structure and nucleus, enabling to distinguish: 1) intact fibers, with oval shape, homogenous structure and peripheral nucleus; 2) regenerative fibers with oval shape and centered single, or polarized multiple nuclei and 3) disintegrated fibers with irregular shape and disintegrated structure (**Supplementary Figure 2A**). Compared to control mice, GW2580 treated mice showed lower numbers of regenerative fibers and significantly higher frequencies of disintegrated fibers, suggesting reduced debris clearance and regeneration with CSF-1 inhibition (**Figure 3D**). This was associated with strong changes in myeloid cell dynamics. The post-ischemic rise in Ly6C^hi^ monocytes in peripheral blood (PB) and muscle, and differentiation of muscle monocyte into ischemic macrophages observed in control mice (7–9) were severely impaired in GW2580 treated mice. Also, the population of Ly6C^lo^ monocytes practically disappeared with GW2580 treatment from all tissue compartments (**Figure 3E**, **Supplementary Figures 3B, C**). In contrast, while initial recruitment of neutrophils was unchanged in GW2580 treated mice, their persistence in ischemic tissue, but not in PB and spleen, was prolonged (**Figure 3E**, **Supplementary Figures 3B, C**), reflecting prolonged inflammation. Notably, F4/80^+^ splenic macrophages were also reduced after GW2580 treatment, underscoring the role of CSF-1 signaling in sustaining resident macrophage populations (**Supplementary Figure 3C**).

In line with reduced frequencies of monocytes and macrophages in iTA and their ability to produce CCL2 (**Supplementary Figure 1D**), expression of *Ccl2* in muscle of GW2580 treated mice 3 days after HLI was significantly reduced (**Figure 3F**).

To characterize monocyte/macrophage phenotype changes resulting from CSF-1 inhibition, we sorted GFP*^+^* CD11b^+^ cells from iTA muscle at d3 as described (**Figure 2A**) and performed gene expression analysis. CSF-1 inhibition resulted in strongly reduced expression of *Ccl2* (**Figure 3G**), which was in line with downregulation of *Ccl2* expression in iTA tissues at the same time point (**Figure 3F**). CSF-1 is known to stimulate glucose uptake by macrophages (43), and glucose uptake is increased in the ischemic muscle, to which Ly6C^hi^ monocytes and macrophages are significant contributors (9). We therefore analyzed expression of genes involved in glucose uptake and metabolism. Treatment with GW2580 significantly reduced expression of metabolic genes *Hk1*, *Pkm1*, *Ldh1* and *Slc2a1*, encoding glucose transporter GLUT1, in GFP*^+^* CD11b^+^ cells (**Figure 3G**), suggesting reduced metabolic activity. Although *Pkm2* was highly expressed, it remained unchanged upon inhibition of CSF-1 signaling. Overall, these data demonstrate impaired metabolic adaptation and reduced migratory activity of monocytes/macrophages with CSF-1 inhibition.

Since GW2580 treatment may have off-target effects on other growth factor receptors we next employed CSF-1 blockade with specific neutralizing antibody injected subcutaneously into the ischemic limb (**Figure 3H**). Compared to control treatment, mice treated with CSF-1-neutralizing antibodies showed slightly increased *Csf1* expression in ischemic muscle (**Supplementary Figure 3D**). Overall, myeloid cell dynamics recapitulated the findings in GW2580 treated mice; namely, strongly reduced numbers of post-ischemic Ly6C^hi^ monocytes and macrophages, systemic reduction of Ly6C^lo^ monocytes, increased *Csf1* expression but reduced expression of *Ccl2* in ischemic muscle of aCSF-1-treated mice (**Figure 3I, J**, **Supplementary Figures 3D–F**). This confirms specificity of the observed effects for CSF-1 inhibition. To conclude, CSF-1 inhibition impairs the differentiation of ischemic macrophages, which is associated with impaired tissue perfusion and muscle regeneration. This indicates that instructive signals for macrophage differentiation play an essential role in perfusion recovery and tissue regeneration after ischemia.

### Dll1 and CSF-1 cooperate in ischemic macrophage instruction.

**Figure 4:**
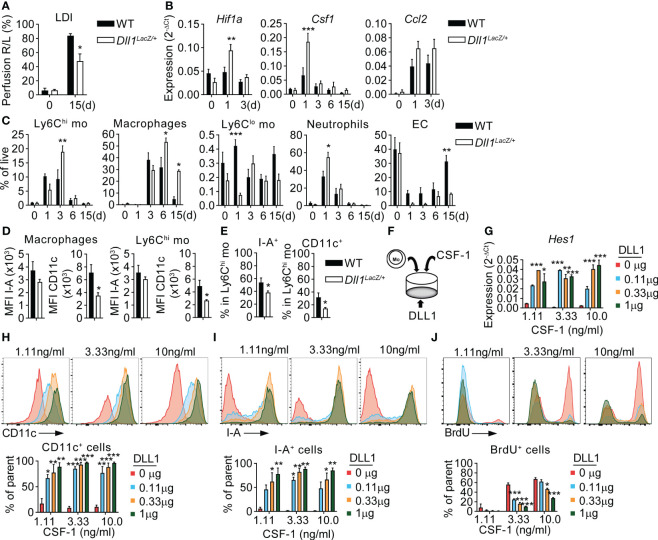
CSF-1 and Notch signaling cooperate in a non-redundant fashion to instruct macrophage cell fate. **(A)** Footpad perfusion measurement in *Dll1^LacZ/+^* mice and WT littermates by LDI, immediately after induction of HLI (d0) and at a d15. **(B–E)** Analysis of TA muscles from *Dll1^LacZ/+^* mice and WT littermate controls before (d0) and after HLI induction. **(B)** Gene Expression analysis from RNA isolated from TA muscles before (d0) and after induction of ischemia. n=4/9/9/3/6 (WT) and n=3/9/10/5/5 (*Dll1^LacZ/+^*). **(C)** Frequencies of myeloid cells and EC, n=4/7/7/6/6 WT and n=3/6/7/8/5 (*Dll1^LacZ/+^*) *P<0.05, **P<0.01, ***P<0.001, 2-way ANOVA with Tukey’s multiple comparison test; (*)differences between WT and *Dll1^LacZ/+^* data. **(D, E)** Phenotype of Ly6C^hi^ monocytes and macrophages at d3 of HLI: **(D)** MFI values of I-A and CD11c in macrophages (left) and Ly6C^hi^ monocytes (right); **(E)** frequencies of I-A^+^ and CD11c^+^ cells in Ly6C^hi^ monocytes. n=4/7/7/6/6 WT and n=3/6/7/8/5 (*Dll1^LacZ/+^*) *P<0.05, unpaired t-test. **(F-J)** Ly6C^hi^ monocytes were sorted from the BM of the *Cx3cr1^gfp/+^* mice and cultured in the presence of different concentrations of plate bound DLL1 and soluble CSF-1. **(F)** culture setup; **(G)** Expression of *Hes1*. RNA was isolated from cells after 48hr of culture. Data are representative of N=3 independent experiments. *P<0.05, **P<0.01, ***P<0.001, 2-way ANOVA with Tukey’s multiple comparison test. **(H, I)** Expression of CD11c **(H)** and I-A **(I)**; **(J)** 16hr BrdU incorporation by GFP^+^CD11b^+^F4/80^+^ cells. **(H–J)** Results are from 72hr of culture, representative flow cytometry analysis (upper panel) and column diagram (lower panel) are shown. Column data are pooled from N=2 experiments. *P<0.05, **P<0.01, ***P<0.001, 2-way ANOVA with Tukey’s multiple comparison test.

Notch signaling regulates macrophage differentiation and maturation from Ly6C^hi^ monocytes during ischemia, which is regulated by Notch ligand Delta-like 1 (Dll1) expressed by arterial EC. In fact, Dll1-deficient mice show impaired arteriogenesis, increased numbers of macrophages with immature differentiation profile and decreased ischemic muscle regeneration (8, 38). Muscle *Dll1* expression is upregulated upon induction of ischemia, reaching its peak at day 3, which coincides with the temporal pattern of ischemic macrophages differentiation from infiltrating Ly6C^hi^ monocytes (8) and the induction of CSF-1 production (**Figures 1E, F**). To test whether Dll1/Notch and CSF-1 have non-redundant functions, we performed experiments in *Dll1^LacZ/+^* haploinsufficient mice (38, 44). Compared to wild-type (WT) controls, *Dll1^LacZ/+^* mice showed prolonged perfusion defects after HLI (**Figure 4A**), as described previously (38). This was associated with increased expression of *Hif1a* and its target gene *Hmox1* at d1 after HLI, consistent with a pronounced hypoxic response (**Figure 4B**, **Supplementary Figure 4A**) (45). Furthermore, Dll1-haploinsufficient mice also showed higher expression of *Csf1* in iTA muscle (**Figure 4B**). This was paralleled by increased cytokine expression (**Supplementary Figure 4B**) and a pronounced cellular inflammatory response (**Figure 4C**). At the same time, numbers of live ECs were significantly and persistently reduced in *Dll1^LacZ/+^* mice, suggesting impaired neoangiogenesis (**Figure 4C**).

Ischemic macrophages express CD11c during maturation *in vivo*, which is associated with ischemic muscle repair (8). Despite higher levels of CSF-1, macrophages in *Dll1^LacZ/+^* mice showed impaired expression of CD11c, but normal expression of the general macrophage marker I-A (MHC class II). These changes were also seen in muscle infiltrating Ly6C^hi^ monocytes, but to a lesser extent (**Figure 4D**). However, Ly6C^hi^ monocytes showed a reduction of I-A^+^ and CD11c^+^ cell frequencies (**Figure 4E**). This indicates intact initial macrophage lineage differentiation, but impaired maturation in the absence of Dll1. Furthermore, changes in monocytes and macrophage phenotypes were reflected in a pro-inflammatory expression profile in the early post-ischemic phase, characterized by significantly increased expression of *Il1b*, *Tnfa*, *Il6* and *Tgfb* in ischemic muscle of *Dll1^LacZ/+^* mice after HLI (**Supplementary Figure 4A**), while no significant difference was found in upregulation of *Ccl2* (**Figure 4B**). Frequencies of DC and Ly6C^lo^ monocytes were identical between the two groups (**Figure 4C**, **Supplementary Figure 4B**) and no differences in cell frequencies in peripheral blood and spleen were found between WT and Dll1 haploinsufficient mice (**Supplementary Figure 4C**). Notably, the number of PDPN^+^ cells and subpopulations was also unchanged in *Dll1*-deficient mice (**Supplementary Figure 4D**).

These data suggest non-redundant function of CSF-1 and Dll1 in instructing or sustaining a mature macrophage phenotype in ischemia. We therefore employed an *in vitro* culture system to study the individual effects of CSF-1 and Dll1 in a defined setting. Ly6C^hi^ monocytes were sorted from the bone marrow of *Cx3cr1^gfp/+^* mice and cultured in control plates or plates pre-coated with different concentrations of recombinant DLL1, in the presence of different concentrations of CSF-1. After 48 or 72hrs of culture, GFP^+^CD11b^+^F4/80^+^ macrophages were collected and analyzed by gene expression analysis and flow cytometry (**Figure 4F**). Gene expression analysis revealed that expression of *Hes1*, a transcriptional target of Notch signaling indicating Notch activation, was dependent on DLL1 concentration, but not CSF-1 concentration (**Figure 4G**). Furthermore, macrophage differentiation markers CD11c and I-A were very low in the absence of DLL1, but both markers became upregulated in a dose dependent manner, while a 10-fold difference in CSF-1 dose did not significantly alter expression of either CD11c or I-A (**Figures 4H, I**), suggesting non-redundant differentiation cues provided by Notch signaling. In contrast, macrophage proliferation was stimulated by CSF-1 in a dose-dependent manner, which was counteracted by increasing amounts of DLL1 (**Figure 4J**). This suggest non-redundant functions for CSF-1 and Notch, with CSF-1 acting as proliferation or survival stimulus for the macrophage lineage, while Notch provides instructive cues for differentiation. Since no cells are obtained in the absence of CSF-1 (data not shown), this also indicates that CSF-1 is required, but not sufficient, for macrophage differentiation.

## Discussion

We here show that ischemia induces rapid production of CSF-1 by a population of stromal cells, most likely fibroblasts expressing PDPN, which is required to sustain monocyte-derived macrophage differentiation, perfusion restoration and ischemic tissue repair. However, in the absence of Notch ligand Dll1, macrophage maturation and ischemic tissue healing is impaired despite high levels of CSF-1. Thus, CSF-1 is not sufficient to instruct a mature macrophage phenotype associated with tissue recovery. Together with our *in vitro* data demonstrating cooperation of CSF-1 and Dll1 in promoting full macrophage differentiation, these data suggest non-redundant functions of CSF-1 and Dll1 in instructing ischemic macrophage fate.

Macrophages are critical cell types of the inflammatory response. Macrophage differentiation is influenced by cues from the local tissue milieu, known as the niche. CSF-1 is a pivotal lineage factor for the myeloid lineage, promoting the cellular differentiation of monocytes and macrophages (31). After induction of hind limb ischemia, CSF-1 production in muscle peaked at day 3, which was paralleled by upregulation of CCL2, recruitment of Ly6C^hi^ monocytes and differentiation of monocyte-derived macrophages. At this time, monocytes and macrophages produced pro-inflammatory cytokines IL-1β, TNF-α, CCL2 and IL-6. While monocytes numbers subsequently decrease, F4/80^+^CD11c^+^ macrophages persist over several days, contributing to the reparative phase. CSF-1 production generally is not limited to one particular cell type and its cellular sources vary between organs and tissues (26–30, 46). In the ischemic muscle, the main source of CSF-1 were PDPN^+^ stromal cells, and, but to a lesser degree, PDPN^+^CD31^+^LEC, as shown for lymph nodes and bone marrow (27, 28). PDPN is expressed by diverse cell populations, such as podocytes, epithelial cells and fibroblasts, including fibroblastic reticular cells (39). In the steady state muscle, PDPN^+^ cells are located in nerves, lymph vessel walls and surrounding blood vessels. Ischemia induced upregulation of PDPN expression on CD45^-^ cells in the interstitial space, but PDPN expression was also detected in CD45^+^ cell infiltrates, which was described previously in models of bacterial infection and human cancer (47–50). The relevance of this finding is unclear, but may be related to migration, cell-cell interaction or lymphangiogensis, as shown previously. Moreover, PDPN^+^CSF-1^+^ cells localized closely to blood vessels, but CSF-1 expression was not observed in nerves (identified as NGFR^+^), which stands in contrast to the situation in intestinal muscle, in which CSF-1 is expressed by neurons (30). Some but not all perivascular PDPN^+^ cells were expressing mesenchymal stromal cell markers PDGFRα and PDGFRβ, but there was no difference between PDGFRα^+^ and PDGFRα^-^ PDPN^+^ cells in *Csf1* expression. Based on a recent transcriptome analysis, skeletal muscle harbors a great number of cells of mesenchymal origin, including fibroblasts, which can be distinguished by expression of *Pdgfra*, *Col1a1*, *Lum*, *Pgfbrb*. Based on this we conclude that CSF-1 is expressed by fibroblasts surrounding large vessel, which are located close to nerve endings, and by small vessel pericytes in the interfiber space (51).

Neutralization of CSF-1 actions, by treatment with specific anti-CSF-1 (aCSF-1) or anti-CSF-1R antibodies or use of cFMS Receptor Tyrosine Kinase inhibitors, is a useful approach to study CSF-1 function and is considered as a therapeutic option for autoimmune diseases, atherosclerosis and cancer in both animal models and clinical trials (52). In our study, application of cFMS Receptor Tyrosine Kinase inhibitor GW2580 (42) strongly reduced numbers of infiltrating Ly6C^hi^ monocytes and developing macrophages in muscle and significantly worsened the post-ischemic course, displayed by prolonged muscle edema and reduced perfusion recovery compared to controls. GW2580 fed mice also showed high numbers of degraded fibers, which confirms a role for macrophages as scavengers of tissue debris in the late reparative phase, as was previously described (13). Also, muscles of GW2580 treated mice showed prolonged infiltration with neutrophils, which is in line with previous data from mice with LPS and thioglycolate-induced inflammation and subsequent aCSF-1R treatment (53). Taken together, we postulate that CSF-1 signaling is critically involved in the acute ischemic phase (associated with Ly6C^hi^ monocyte recruitment) and subacute ischemic phase (monocyte-derived macrophage differentation), which is a prequel to resolution and recovery.

Inhibition CSF-1 signaling also had strong, systemic effects on the population of Ly6C^lo^ patrolling monocytes. Ly6C^lo^ monocyte numbers were significantly reduced in peripheral blood, which confirms previous results (53), but also in spleen, which emphasized the critical role of CSF-1 for this monocyte subtype.

Furthermore, inhibition of CSF-1 signaling not only influenced myeloid cell population dynamics, but also affected myeloid cell metabolism. By multimodal PET-CT scanning with radiolabeled glucose we have shown previously that glucose uptake is increased in the ischemic muscle during the acute and subacute phases, to which Ly6C^hi^ monocytes and macrophages are significant contributors (9). Hypoxia is a known inducer of glucose transporters and glycolytic enzymes (54), while CSF-1 stimulation promotes glucose uptake by macrophages (43). CSF-1 signaling inhibition reduced expression of glycolysis-associated genes *Slc2a1*, *Hk1*, *Pkm1* and *Ldh1*, indicating reduced metabolic activity of macrophages (16). These transcriptional changes may translate into reduced proliferation of monocytes and macrophages and at least partially explain the reduced numbers of macrophages associated with impaired muscle regeneration, since the glycolytic switch is often a prerequisite for proliferation (13, 43, 55). Based on this, we conclude that CSF-1 mediates the adaptation of monocytes and macrophages to ischemia.

Our results also demonstrate a link between CSF-1 and the chemokine CCL2, a mediator of inflammatory monocytes recruitment to muscle (7, 8). CSF-1 has direct effects on the macrophage actin cytoskeleton, namely membrane ruffling, stimulation of lamellipodial protrusions and remodeling of actin cytoskeleton with subsequent polarization (24, 25, 56). However, we found that besides EC, LEC and PDPN^+^ stromal cells, recruited monocytes and macrophages also expressed significant amounts of *Ccl2*, while treatment with GW2580 significantly reduced *Ccl2* expression in monocytes/macrophages as well as in tissues. These results suggest that the migratory effects of CSF-1 are at least in partially mediated through stimulation of CCL2 production, which triggers migration in an autocrine manner. The underlying molecular mechanism of CSF-1-induced *Ccl2* expression are still unknown. However, an involvement of the Akt-phosphatidylinositol 3-kinase (PI3K) pathway seems possible, since both are involved in CSF-1 dependent survival and proliferation of macrophages (57, 58) and were recently suggested to control CCL2 production and migration of tumor-associated macrophages in breast cancer patients (59).

Finally, our data demonstrate a requirement for cooperation of CSF-1 and Notch signaling, mediated by Notch ligand Dll1, for full functional differentiation of macrophages in the ischemic niche. Notch signaling is a cell contact-dependent regulator of terminal differentiation and function of resident tissue macrophages (35–37), but also monocyte-derived macrophages (8). Notch signaling often occurs in specific niches, where it may also regulate other niche resident cells, e. g. PDGFRα^+^ fibroblasts (35). The ligand Dll1 is specifically expressed by arterial EC (8, 38) and its expression is strongly upregulated after induction of hind limb ischemia (8, 38). Furthermore, Notch signaling activated by endothelial Dll1 is a major driver of ischemic macrophage maturation and terminal differentiation (8). In contrast to WT mice, *Dll1* haploinsufficient mice showed higher and more persistent numbers of Ly6C^hi^ monocytes and macrophages, but phenotypic analysis revealed an immature differentiation profile, e. g. reduced expression of CD11c (8). This was accompanied by higher levels of CSF-1 and increased levels of *Hif1a* and pro-inflammatory cytokines in macrophages, reflective of more severe ischemia and unrestrained inflammation. HIF-1α expression, however, was not verified by protein staining. These findings were extended in an *in vitro* culture system, were CSF-1 induced macrophage proliferation (and survival), while Dll1-dependent Notch signaling limited proliferation and promoted macrophage differentiation.

Our data on the cooperation of CSF-1 and Notch also highlight the cellular and molecular complexity of niche signaling. In our model, arterial-endothelial Dll1 and PDPN^+^ fibroblast-derived CSF-1 mediate differentiation and functional maturation of Ly6C^hi^ monocytes in a non-redundant manner. Unlikely previous report (35), we did not find evidence of reduced -stromal cell numbers in *Dll1* haploinsufficient mice, maybe related to the spatiotemporal expression pattern of Dll1 precluding cell-to-cell contact in the stromal niche. In fact, our model clearly points to a major role of endothelial Dll1 in this scenario. However, this does not rule out redundant or non-redundant actions of other Notch ligands, such as Dll4, which is expressed by capillary/microvascular EC (8, 38), or Jag1 expressed by stromal or recruited cells. Taken together, our data suggest that CSF-1 is required but not sufficient to induce functional ischemic macrophage differentiation, and that the cooperation with Dll1 is required to induce full functional maturation. Both actions need to cooperate to promote a macrophage phenotype capable to promote angiogenesis and tissue repair. From this it would follow that a therapeutic concept for patients with peripheral limb ischemia based solely on providing CSF-1 growth factor would fail in the absence of a proper set of instructive cues, involving Notch ligands presented by vascular EC, which may be impaired in chronic vascular disease.

## Materials and methods

### Mice

*Cx3cr1^gfp/+^(B6.129P2(Cg)-Cx3cr1^tm1Litt^/J)* (60) (C57BL/6 background) and *Dll1^LacZ/+^ (129-Dll1^tm1Gos^/J)* (SV129 background) (44) mice had been described previously. 10-12 weeks old male mice were used for the experiments. Animals were housed under specific pathogen-free conditions at 14/10hr light/dark cycle and free access to standard lab animal diet (Altromin) and autoclaved tap water. All experiments were approved by local animal welfare authorities of Hannover Medical School and Lower Saxony (LAVES).

### Hind limb ischemia

Experiments with hind limb ischemia were carried out as described (8, 9, 61). Briefly, mice were anesthesized through intraperitoneal injection of Ketamin (80mg/kg, CPPharma), Xylavet (Xylazin 2.5mg/kg, CPPharma) and Dormicum (Midazolam 2.5mg/kg, Ratiopharm), diluted in 0.9% NaCl (Braun), 5μl per 1 gram of body weight. After a right inguinal incision, the neurovascular bundle was exposed under microscopic control and the superficial branch of the femoral artery was surgically ligated distal to the origin of the deep femoral branch. To avoid post-operative hypothermia, mice were maintained on heating pads until they had been fully awake. Perfusion was measured by Laser Doppler Imaging (LDI) of plantar regions of interests with Perimed LDPI PIM II Laser Scanner (Perimed, Sweden). Animals with less than 90% relative perfusion reduction post-surgery were excluded from the study. Experimenters were unaware of treatment allocation or genotype.

### Animal treatment

GW2580 (LC Laboratories) was administered once daily by oral gavage at a dose of 80 mg/kg in 0.1% Hydroxipropylmethylcellulose/0.1% Tween-20 as described previously (62). Treatment started 4 days prior HLI induction. Body weight was measured daily to exclude weight loss. Anti-CSF-1 (aCSF-1) antibody treatment (Clone 5A1, BioXCell) or PBS control was subcutaneously injected in the ischemic limb, at a dose of 50 μg, immediately after surgery or up to 3 days (for details see **Figure 3H**).

### Cell isolation

Spleens were pressed, resuspended in PBS (Sigma) and filtered through 70um mesh (Nitex). Blood samples were filtered as indicated. Red blood cells from spleen and blood samples were removed through treatment with RBC lysis buffer (Biolegend) and subsequent washing with PBS. *M. Tibialis anterior* and *M. semimembranosus* were excised, small piece was snap-frozen in the liquid nitrogen. The rest of the tissue was minced and incubated in DMEM (Sigma) containing 500U/ml type 2 collagenase (Worthington) and 1% penicillin/streptomycin (Sigma) for 22 min at 37^0^C. Then, tissue was disintegrated using GentleMACS dissociator (Miltenyi Biotec, program “spleen 01_01”) and the incubation step was repeated for another 22 min. Finally, tissue lysate was resuspended, filtered as indicated above and cells were washed with PBS. Bone marrows were flushed from the long bones through spinning at 5000g, 30sec, filtered twice and resuspended in PBS. For sorting purposes, single cell suspension was overlaid on equal amount of Histopaque 1083 (Sigma), spun at 430g 25min. at room temperature (RT), interphase was collected and washed with PBS. Cell suspensions from all organs were counted using Countess II FL automated cell counter (Invitrogen). Dead cells were excluded through 0.4% trypan blue (Sigma) staining.

### Flow cytometry and cells sorting

Cells were resuspended in staining buffer containing PBS supplemented with 2% fetal calf serum (FCS, Biochrom), 2mM Na_2_EDTA (Roth) and 0.02% NaN_3_ (AppliChem). 0.5-1x10^6^ cells were plated and stained with antibodies and streptavidin compounds, listed in **Supplementary Table S1.** Propidium Iodide (Fluka) was used to exclude dead cells. Flow cytometric analysis was performed using LSR II (BD biosciences; acquisition software BD FACSDiva Software v8.0.1). BD FACSAria™ Fusion Flow Cytometer was used for cell sorting. All flow cytometry data were analyzed using FlowJo software v8.0.1 (TreeStar). Cell definition is summarized in **Table 1**.

### *In vitro* cell culture

12-well cell culture plates (TPP) were pre-coated with different amounts of chimeric mouse DLL1-Fc protein or Fc fragment alone (both from R&D) dissolved in PBS, at RT for 3hr. Single cell suspensions were prepared from the bone marrows of *Cx3cr1^gfp/+^* mice, stained for CD11b and Ly6C and GFP^+^CD11b^+^Ly6C^hi^ monocytes were sorted as described above. 1.6x10^5^ cells were plated in pre-coated wells, in RPMI-1640 medium (BioCell), supplemented with 10%FCS (Biochrom), 1% Glutamax (Gibco) and 1% Penicilin-Streptomycin (Biochrom), in the presence of different concentrations of recombinant mouse M-CSF (Peprotech). After 48hr of culture, medium was supplemented with the new dose of M-CSF, equal to initial amounts. Finally, cells were collected after 72hr of culture and used for flow cytometry analysis or RNA isolation. To quantify proliferation, BrdU (Sigma) was added to culture medium at a 10μM final concentration, during the last 16hr of 72hr culture and incorporation was detected by flow cytometry, using BrdU flow kit (BD Pharmingen), according to manufacturer’s instructions.

### Cytokine bead array

CBA analysis was performed with serum and muscle extract samples. Blood was collected without anticoagulant. After clot formation, samples were spun at 1000g, 10min. and serum was harvested. TA muscles were excised, minced and immediately placed in the ice-cold extraction buffer: 0.05% Tween-20 (BioRad), 0.1% fraction V BSA (Roth) in PBS, supplemented with complete mini protease inhibitor cocktail (EDTA-free, 1 tablet per 10ml; Roche). Then, tissue was mechanically homogenized and incubated on ice for 30min. Finally, samples were vortexed, spun at 17000g for 20min., 4^0^C and supernatants were collected.

CBA was performed using LEGENDPlex™ Mouse HSC Myeloid panel kit (Biolegend), according to manufacturer’s instructions. Samples were acquired on LSR II flow cytometer (see above). Results were analyzed using LEGENDPlex™ online software (Biolegend), according to manufacturer’s instructions. Concentrations in muscle were normalized on tissue mass and extraction volume.

### Histology and immunohistochemistry

Histologic and Immunohistochemistry analysis of muscle tissue was performed as previously described (61) with modifications. Tissues were fixed in 4% PBS-buffered paraformaldehyde (PFA, Sigma) and embedded in paraffin or cryopreserved in increasing concentrations (15% and 30% in PBS) of sucrose (Roth), and embedded in Tissue-tek OCT compound (Sakura). Blocks were sectioned with a rotation microtome (Leica) into 2µm (paraffin blocks) or 8 µm (frozen blocks) slices. For morphology analysis, paraffin sections were deparaffinized and stained with H&E according to routine protocol. To detect CSF-1, antigen retrieval procedure was performed on deparaffinized sections, by boiling in 10mM Citrate (Sigma) buffer pH6.0 for 16min. and subsequent cooling on ice. Blocking buffer was prepared using appropriate sera from the hosts of secondary antibodies, at 10% final concentration in 3%BSA/PBS. Fc blocking reagent TrueStainX anti-CD16/32 (Biolegend) was used if needed. For intracytoplasmic markers, fixation-permeabilization was performed using 0.15% Triton-X100 (Roth)-containing PBS respectively. After antibody staining, nuclei were counterstained with DAPI (Roth) and sections were embedded in the fluorescence mounting medium (DAKO). Primary and secondary antibodies used for IHC are listed in **Supplementary Table S2**. Light microscopy was performed using Leica DFC425 C microscope, images were acquired and processed using Leica Application Suite v. 3 software. Confocal images were acquired using Leica DMi8 (Leica Microsystems) inverted microscope, with x20 immersive objective. Data were processed using Las AF Lite Software (Leica Microsystems). Sizing of microscopy images was performed using Adobe Illustrator (Adobe).

For quantification analysis of H&E stained samples, morphology of fibers was evaluated according to their shape and architecture and location of nuclei. Total amount of fibers were counted per section and % of each group: intact, regenerative or disintegrated fibers was determined based on it (for details see **Supplementary Figure 2A**).

### RNA isolation and real time PCR

Total RNA was purified using Nucleospin RNA plus Kit (Macherey Nagel) according to manufacturer’s instructions. Qualitative and quantitative analysis was performed using Nanodrop 2000 (Thermofisher scientific) spectrophotometer. Then, RNA was transcribed into cDNA using cDNA synthesis kit (Invitrogen) and quantitative real-time PCR was performed with Fast Start Essential DNA Green Master Mix on a LightCycler 96 system (both from Roche). All procedures were run according to the manufacturer’s instructions. The expression of each specific gene was normalized to the expression of housekeeping *Rps9*, using 2^-^<σπ>Δ</σπ>^Ct^ method. Primer sequences are listed in the **Supplementary Table S3**.

### Statistics

Results were analyzed with GraphPad Prizm 9 software. All data are expressed as mean ± SEM. Difference between groups were determined using 2-way ANOVA with Tukey’s and Bonferroni’s multiple comparison test, 1-way ANOVA with Dunnett’s multiple comparison test, Mann-Witney’s or 2-tailed unpaired t-test. *P<0.05* was considered to be statistically significant difference.

## Data availability statement

The original contributions presented in the study are included in the article/**Supplementary Material**. Further inquiries can be directed to the corresponding author.

## Ethics statement

The animal study was approved by local animal welfare authorities of Hannover Medical School and Lower Saxony (LAVES). The study was conducted in accordance with the local legislation and institutional requirements.

## Author contributions

TK designed the study, performed experiments, collected, analyzed and interpreted the data, prepared the figures and wrote the manuscript draft. SS, DK and JG performed experiments, HH and KS-O provided approval of the final version, FL designed the study, provided resources, supervised research and wrote the manuscript. All authors contributed to the manuscript and approved the final version.
