# Supplementary material for: CSF-1 and Notch signaling cooperate in macrophage instruction and tissue repair during peripheral limb ischemia
Source: Front Immunol. 2023 Aug 24;14:1240327. doi: 10.3389/fimmu.2023.1240327 (PMC10484478; doi:10.3389/fimmu.2023.1240327)
Supplement: Supplementary file 1 [file DataSheet_1.pdf]

Supplementary Material

CSF-1 and Notch signaling cooperate in macrophage instruction and tissue repair during peripheral limb ischemia

Tamar Kapanadze[1,2], Jaba Gamrekelashvili[1,2], Stefan Sablotny[1,2], Dustin Kijas[1,2], Hermann Haller[2], Kai Schmidt-Ott[2], Florian P. Limbourg[1,2].

[1]Vascular Medicine Research, Hannover Medical School, 30625 Hannover, Germany.
[2]Department of Nephrology and Hypertension, Hannover Medical School, 30625 Hannover, Germany

Correspondence: Florian P. Limbourg: Limbourg.Florian@mh-hannover.de

Supplementary Figures

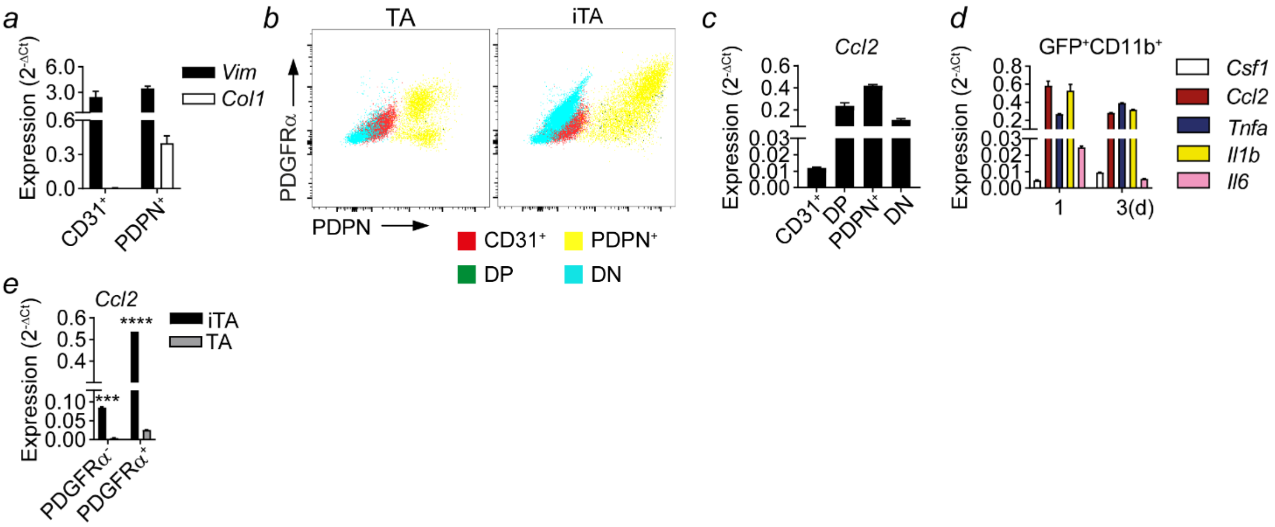

Supplementary Figure 1. **Characterization of ischemic cell populations changes.** (*a*) Expression of *Col1* and *Vim*: RNA was isolated from CD45$^-$CD31$^+$ and CD45$^-$CD31$^-$PDPN$^+$ cells, sorted from iTA at d1. Data are pooled from N=3 independent experiments. (*b*) Expression of PDGFRα: Representative flow cytometry analysis of different CD45$^-$ cell populations of iTA and TA at d1 after HLI. (*c*) Expression of *Ccl2*. RNA was isolated from different CD45$^-$ cell populations sorted from iTA muscles at d1. Data are a representative of N=3 independent experiments. (*c*) Expression of cytokines and chemokines, RNA was isolated from CD11b$^+$GFP$^+$ cells, sorted from iTA muscles of *Cx3cr1*$^{gfp/+}$ mice at d1/d3 of HLI. Representative of N=2 independent experiments. (*d*) Expression of *Ccl2*, RNA was isolated from PDGFRα$^+$ and PDGFRα$^-$ cells, sorted from iTA and TA muscles at d1 after HLI. Data are representative from N=2 independent experiments, ****P<0.001*, *****P<0.0001*, Unpaired t-test.

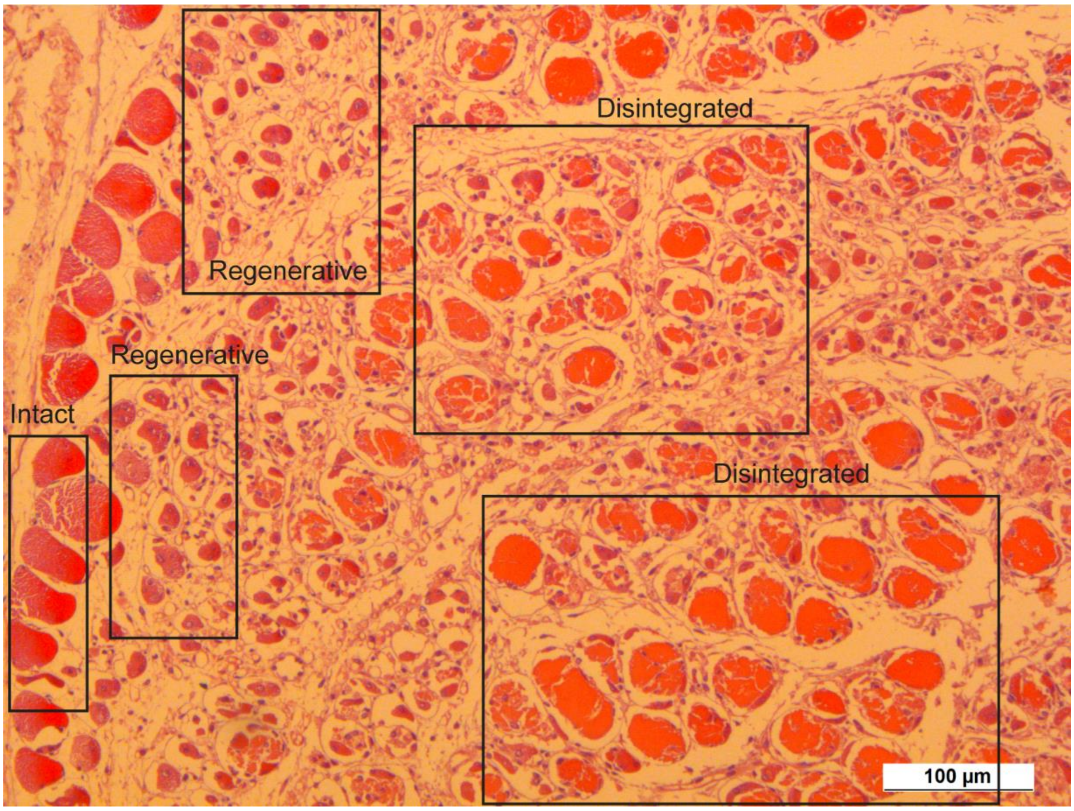

Supplementary Figure 2: Ischemia affects muscle fibers (a) H&A staining of paraffin-embedded section of iTA, d6 after HLI. 1) Intact fibers: oval shape, homogenous structure and peripheral nucleus; 2) regenerative fibers: oval shape and centered single, or polarized multiple nuclei and 3) disintegrated fibers: irregular shape and disintegrated structure.

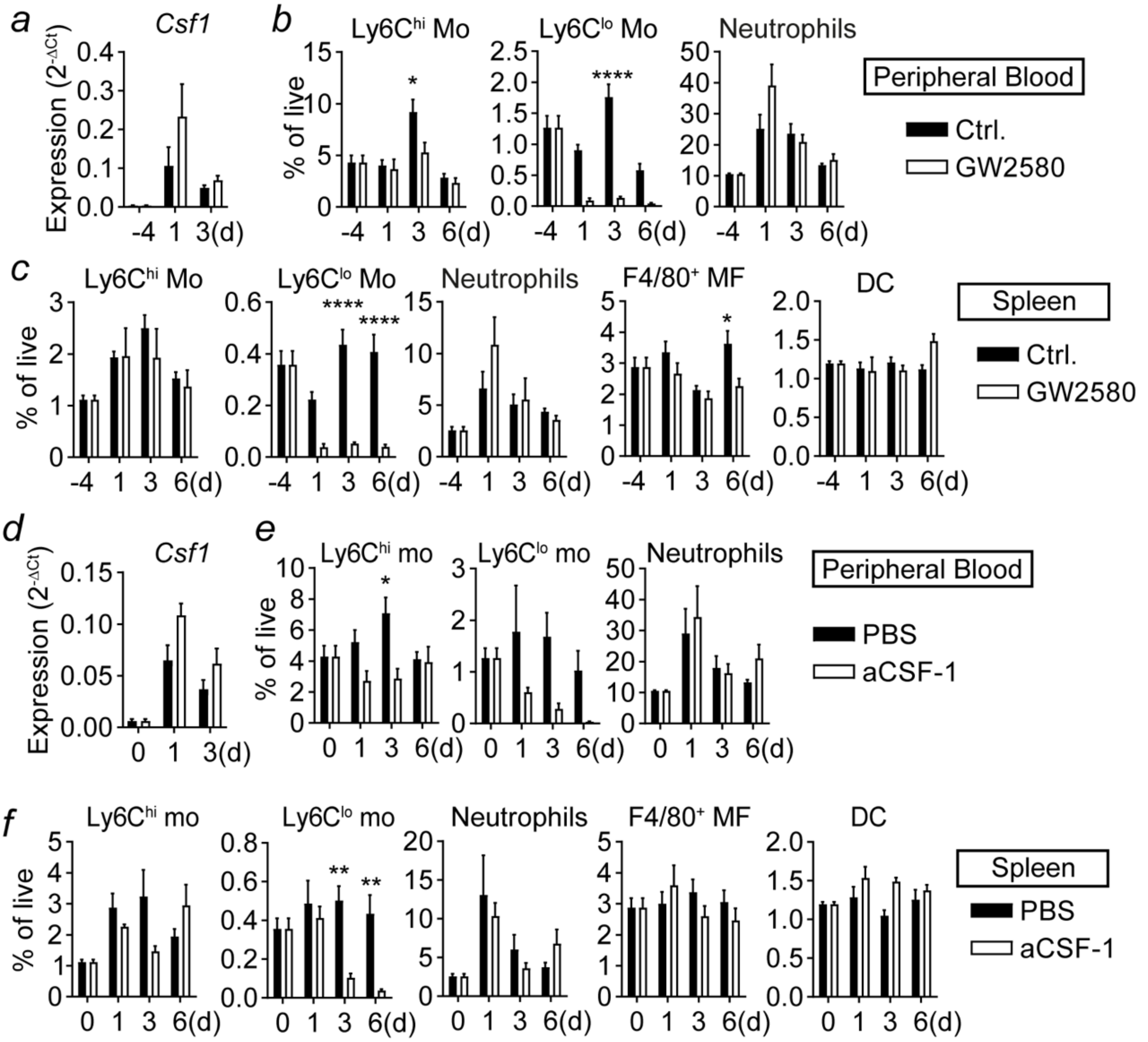

Supplementary Figure 3. **Local and systemic effect of CSF-1 blockade** (*a-c*) Administration of CSF-1 signaling inhibitor GW2580 in *Cx3cr1*$^{gfp/+}$ mice. Data show: (*a*) Expression of *Csf1*. RNA was isolated from TA muscle, n=3/3/9 (ctrl.) and n=3/4/7 (GW2580) and (*b-c*) frequencies of myeloid cells in the peripheral blood (*b*) and spleen (*c*) of ctrl. and GW2580-fed mice. n=3/6/11/5 (ctrl.) and n=3/5/6/6 (GW2580). **P<0.05, ****P<0.0001.* 2-way ANOVA with Tukey's multiple comparison test. (*d-f*) Treatment of mice with neutralizing aCSF1 antibody. Data show: (*d*) Expression of *Csf1*. RNA was isolated from TA muscle, n=3/3/5 (both groups) and (*e-f*) frequencies of myeloid cells in the peripheral blood (*e*) and spleen (*f*) from mice treated with PBS or aCSF-1, after induction of HLI. n=4/3/5/4 (ctrl.) and n=4/3/5/6 (aCSF-1) mice; **P<0.05, ***P<0.001,* 2-way ANOVA with Tukey's multiple comparison test. (*d-f*) d0 corresponds to not operated mice.

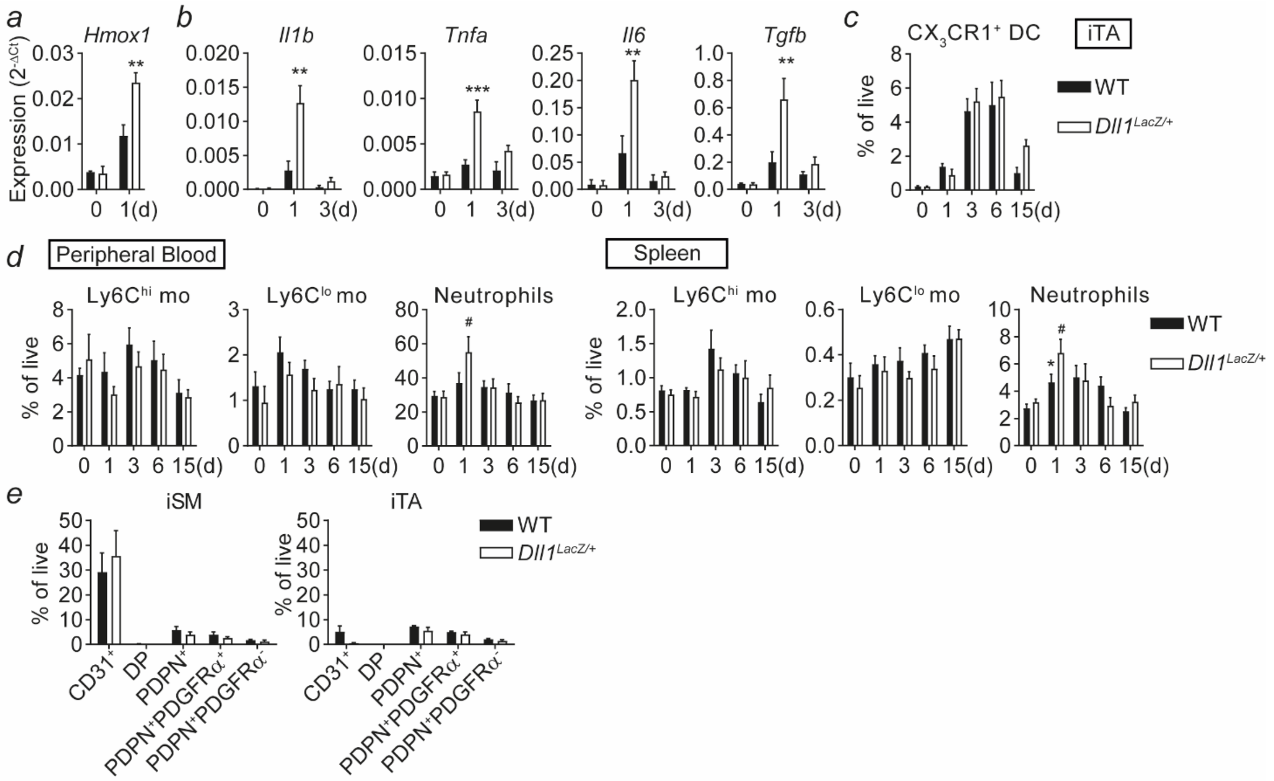

Supplementary Figure 4. **Inflammatory response in *Dll1$^{LacZ/+}$* mice.** (*a*, *b*) Gene expression analysis. RNA was isolated from TA muscle before (d0) or after induction of HLI. (a) HIF-1α target gene *Hmox1*, *** P=0.003* WT (n=4/7) vs *Dll1$^{LacZ/+}$* (n=3/8). 2-way ANOVA with Tukey's multiple comparison test. (b) *Tgfb* and *Il6*: n=4/9/9 (WT) and n=3/9/10 (*Dll1$^{LacZ/+}$*); *Il1b* and *Tnfa*: n=3/7/3 (WT) and n=2/6/3 (*Dll1$^{LacZ/+}$*). ***P<0.01, ***P<0.001.* 2-way ANOVA with Tukey's multiple comparison test, WT vs. *Dll1$^{LacZ/+}$*. (*c*) DC frequencies in iTA muscles from *Dll1$^{LacZ/+}$* mice and WT littermate controls, n=4/7/7/6/6 WT and n=3/6/7/8/5 (*Dll1$^{LacZ/+}$*). (*d*) Cell frequencies in the peripheral blood (left) and spleen (right), before (d0) and after induction of HLI. n=6/7/7/6/6 (WT) and 6/6/7/8/5 (*Dll1$^{LacZ/+}$*). *$^{*/\#}$P<0.05*, difference to d0. (*e*) Frequencies of different CD45$^-$ cells in iSM and iTA muscles at d3 after induction of HLI.

Supplementary Table 1: List of antibodies used for flow cytometry.

Antibody	Clone	Label	Dilution	Company	Cat. #
CD32/16	93	Unlabeled	1:200	Biolegend	101319
CD45	30-F11	AF700	1:400	Biolegend	103128
F4/80	BM8	APC	1:100	Biolegend	123116
F4/80	BM8	BV650	1:100	Biolegend	123149
CX3CR1	SA011F11	PE	1:200	Biolegend	149005
CD115	AFS98	AF488	1:200	Biolegend	135511
CD115	AFS98	APC	1:200	Biolegend	135509
CD117	2B8	APC-Cy7	1:100	Biolegend	105803
CD19	6D5	Bio	1:400	Biolegend	115504
B220	RA3-6B2	Bio	1:400	Biolegend	103203
CD3	17A2	Bio	1:200	Biolegend	100243
Ter119	Ter119	Bio	1:400	Biolegend	116203
NK1.1	PK136	Bio	1:200	Biolegend	108704
Ly6G	1A8	Bio	1:400	Biolegend	127603
CD11b	M1/70	Pacific Blue	1:400	Biolegend	101224
Ly6C	HK1.4	PE-Cy7	1:1400	Biolegend	128018
I-A/I-E	M5/114.15.2	BV510	1:400	Biolegend	107635
CD11c	N418	BV605	1:400	Biolegend	117334
CD43	S7	PerCP-Cy5.5	1:400	BD Pharmingen	562865
CD31	390	FITC	1:100	Serotec	MCA1364F
CD31	390	AF647	1:400	Biolegend	102416
CD140a	APA5	Bio	1:100	Biolegend	135909
CD140a	APA5	BV605	1:200	Biolegend	135916
BrdU	3D4	AF647	1:100	BD Biosciences	560209
Podoplanin	8.1.1.	PE	1:400	Biolegend	127408
Streptavidin		APC	1:800	Biolegend	405207
Streptavidin		APC-Cy7	1:400	Biolegend	405208
Streptavidin		PE-Dazzle 594	1:400	Biolegend	405248

Supplementary Table 2: List of antibodies used for IHC.

Marker	Host/Isotype	Target	Clone	Label	Dilution	Producer
CD32/16	Rat IgG2a, λ	Mouse	93	-	1:200	Biolegend
CD45	Rat IgG2b, κ	Mouse	30-F11	FITC	1:100	BD Pharmingen
Podoplanin	Hamster IgG1	Mouse	8.1.1.	PE	1:400	Biolegend
Podoplanin	Hamster IgG1	Mouse	8.1.1.	-	1:200	Novus Biologicals
α-SMA	Mouse monocl.	Mouse	1A4	AF488	1:200	Invitrogen
CD31	Rat IgG2a	Mouse	390	AF647	1:400	Biolegend
CD31	Rat IgG2a	Mouse	SZ31	-	1:20	Dianova
CX_3CR1	Mouse monocl.	Mouse	SA011F11	PE	1:200	Biolegend
CD140a	Rat IgG2a	Mouse	APA5	Biotin	1:100	Biolegend
CD140b	Rat IgG2a	Mouse	APB5	-	1:100	eBioscience
F4/80	Rat IgG2a	Mouse	BM8	APC	1:100	Biolegend
LYVE-1	Goat IgG	Mouse		Biotin	1:200	R&D Systems
NGFR (p75)	Goat IgG	Mouse		-	1:200	R&D Systems
CSF-1	Rabbit IgG	Human, Mouse		-	1:100	BosterBio
	Goat	Rat		Cy5	1:200	Jackson Imm.
	Goat	Hamster		Cy3	1:200	Jackson Imm.
	Goat	Rabbit		FITC	1:200	Jackson Imm.
	Donkey	Goat		AF647	1:200	Invitrogen
	Streptavidin	Biotin		APC	1:400	Biolegend

Supplementary Table 3: List of murine primers.

		5'→3'
Csf1	Forward	CCC ATA TTG CGA CAC CGA A
	Reverse	AAG CAG TAA CTG AGC AAC GGG
Hes1	Forward	CCG GAC AAA CCA AAG ACG GC
	Reverse	GGA ATG CCG GGA GCT ATC TTT CT
Ccl2	Forward	TGG GCC TGC TGT TCA CAG TT
	Reverse	TGG GGC GTT AAC TGC ATC TGG
Tnfa	Forward	TAC TGA ACT TCG GGG TGA TTG GTC C
	Reverse	CAG CCT TGT CCC TTG AAG AGA ACC
Il6	Forward	ATC CAG TTG CCT TCT TGG GAC TGA
	Reverse	TAA GCC TCC GAC TTG TGA AGT GGT
Il1b	Forward	AGT TGA CGG ACC CCA AAA G
	Reverse	AGC TGG ATG CTC TCA TCA GG
Tgfb	Forward	GTA CAG CAA GGT CCT TGC CCT
	Reverse	TAG TAG ACG ATG GGC AGT GGC
Pkm1	Forward	TTG TGC GAG CCT CCA GTC
	Reverse	ACT CCG TGA GAA CTA TCA AAG C
Pkm2	Forward	CAT TAC CAG CGA CCC CAC AG
	Reverse	GAG CAC TCC TGC CAG ACT TG
Hk1	Forward	CGG AAT GGG GAG CCT TTG G
	Reverse	GCC TTC CTT ATC CGT TTC AAT GG
Ldha	Forward	TGT GGC AGA CTT GGC TGA GA
	Reverse	CTG AGG AAG ACA TCC TCA TTG ATT C
Slc2a1	Forward	CCA CCA CAC TCA CCA CGC TT
	Reverse	CAT GGA GTT CCG CCT GCC AA
Pdgfra	Forward	TCC TTC TAC CAC CTC AGC GAG
	Reverse	CCG GAT GGT CAC TCT TTA GGA AG
Pdgfrb	Forward	GTG GTC CTT ACC GTC ATC TCT C
	Reverse	GTG GAG TCG TAA GGC AAC TGC A
Hmox1	Forward	GCC GAG AAT GCT GAG TTC ATG
	Reverse	TGG TAC AAG GAA GCC ATC ACC
Vim	Forward	TCC AGC AGC TTC CTG TAG GT
	Reverse	CCC TCA CCT GTG AAG TGG AT
Col1	Forward	TGT CCC AAC CCC CAA AGA C
	Reverse	CCC TCG ACT CCT ACA TCT TCT GA
Hif1a	Forward	TCA TCA GTT GCC ACT TCC CCA C
	Reverse	CCG TCA TCT GTT AGC AC CAT CAC
Rps9	Forward	GGA TTT CTT GGA GAG GCG GC
	Reverse	ACC TGC TTG CGG ACC CTA AT
